# Calendar

**DOI:** 10.1155/S0962935196000440

**Published:** 1996-09


					Calendar
Calendar
1996
4-7 September 1996
2nd International Congress on
Phagocytes: Biology, Physiology,
Pathology and Pharmacotherapeutics,
Pavia, Italy.
Contact: Prof. Giovanni Ricevuti,
Institute of Medical Therapy,
OSM- 27100, Pavia, Italy.
Fax: (+ 39) 382 526341.
22-27 September 1996
ICP '96
10th International Conference
on Prostaglandins and
Related Compounds,
Vienna, Austria.
Contact: ICOS Congress
Organisation Service Ges.m.b.H.,
Johannesgasse 14,
A-1010 Vienna, Austria.
Fax: (+43) (1) 512 80 91 80.
3-4 October 1996
Receptors in Cardiovascular Diseases as
Drug Targets,
Paris, France.
Contact: L. Drye, Institut Pasteur
Eurf/conferences, 28 rue du Docteur
Roux 75724, Paris Cedex 15, France.
Fax: (+ 33) (1) 40 61 34 05.
27-31 October 1996
New Drugs for Inflammatory,
Allergic, and Immunologic Disease:
A Symposium featured at the
8th International Conference on
Inflammation Research,
Hershey, PA, USA.
Contact: Dr J.B. Summers,
Abbot Laboratories,
100 Abbott Park Road,
D47J AP10, Abbott Park,
IL 60064, USA.
Fax: + 1) 708 938 5034.
30 October-1 November 1996
Inflammatory Bowel Diseases From
Bench to Bedside,
Falk Symposium No. 96.
Freiburg, Germany.
Contact: Prof. Dr H. Goebell,
Med. Universitlitsldinik, Essen,
Hufelandstr. 55,
D-45147 Essen, Germany.
Fax: (+ 49) 241 7234694.
21-22 November 1996
BIRAs Meeting: Is there any point in
developing an anti-rheumatoid arthritis
drug? National Heart and Lung Institute,
London, UK.
Contact: Mrs Lin Wells, Bone and Joint
Research Unit, The London Hospital
Medical College, 25-29 Ashfield Street,
London E1 2AD, UK.
Fax: 0171 377 7763.
1-5 December 1996
Inaugural Conference of the Federation of
Immunological Societies of Asia--Oceania
(FIMSA) in conjunction with the 24th
Annual Scientific Meeting of the
Australasian Society for Immunology
(ASI), Adelaide, Australia.
Contact: FIMSA 96 Conference Secretariat,
PO Box 153, Nairne, SA, Australia, 5252.
Fax: 61 8 388 6164.
4-6 December 1996
New Trends in Atherosclerosis Research,
Paris, France.
Contact: L. Drye, Institut Pasteur
Eurf/conferences, 28 rue du Docteur
Roux 75724, Paris Cedex 15, France.
Fax: (+33) (1) 40 61 34 05.
5-8 December 1996
16th European Workshop on
Inflammation: Inflammation and Skin,
Leipzig, Germany.
Contact: Prof. Dr M. Kletzmann,
Institut ffir Pharmakologie, Pharmazie und
Toxikologie, Veteriniirmedizinische
Fakultfit, Universitt Leipzig,
Zwickauer Str. 55,
D-04103 Leipzig, Germany.
Fax: (+49) 341 9738149.
or
Prof. Dr G. Wozel
Klinik ffir Hautkrankhelten,
Medizinische Akademie Carl Gustav Carus,
Fetscherstr. 74, D-01307 Dresden,
Germany.
Fax: (+ 49) 351 4584338.
1997
16-20 November 1997
The 3rd World Congress
on Inflammation,
Tokyo, Japan.
Contact: The Secretariat,
c/o PMSI Japan Ltd,
Royal Bldg 12-8 Nibancho,
Chiyoda-ku, Tokyo 102, Japan.
Fax: (+ 81) 3 5275 6994.
19-22 November 1997
EULAR--50th Anniversary of The
European League Against Rheumatism Xth
Symposium. Vienna, Austria.
Contact: Maria Hamata, MAW, Maria
Rodler & Co Ges.m.b.H., Freyung 6,
A-1014 Vienna, Austria.
Fax: +43) (1) 222 535 60 16.
3-5 December 1977
BSI Fifth Annual Congress, Brighton, UK.
Contact: BSI, Triangle House, Broomhill
Road, London SW18 4HX, UK.
Fax: 0181 877 9308.
1998
20-25 April 1978
5th International Conference on Systemic
Lupus Erythematosus, Cancun, Mexico.
Further information from: Dr Donato
Alarc6n Segovia, Director General,
Instituto Nacional de la Nutricion,
Salvador Zubiran, Vasco de Quiroga 15,
Delegaci6n Tlalpan, C.P. 14000 Mexico
D.F.
Fax: (525) 573 2096.
16-18 December 1998
BSI Sixth Annual Congress, Harrogate,
UK.
Contact: BSI, Triangle House, Broomhill
Road, London SW18 4HX, UK.
Fax: 0181 877 9308.
(C) 1996 Rapid Science Publishers Mediators of Inflammation Vol 5 1996 299

				

